# Lycopene-Rich Extract from Red Guava (*Psidium guajava* L.) Decreases Plasma Triglycerides and Improves Oxidative Stress Biomarkers on Experimentally-Induced Dyslipidemia in Hamsters

**DOI:** 10.3390/nu11020393

**Published:** 2019-02-13

**Authors:** Ana Karolinne da Silva Brito, Geovanni de Morais Lima, Luciana Melo de Farias, Lays Arnaud Rosal Lopes Rodrigues, Vanessa Brito Lira de Carvalho, Cristian Francisco de Carvalho Pereira, Karoline de Macedo Gonçalves Frota, Airton Mendes Conde-Júnior, Ana Mara Oliveira Silva, Márcia dos Santos Rizzo, Clarisse Maria Barbosa Fonseca, Rayane Carvalho de Moura, Raimunda Cardoso dos Santos, José Roberto de Souza de Almeida Leite, Marcos Antônio Pereira dos Santos, Paulo Humberto Moreira Nunes, Daniel Dias Rufino Arcanjo, Maria do Carmo de Carvalho e Martins

**Affiliations:** 1 Medicinal Plants Research Center, Federal University of Piaui, Teresina, PI 64.049-550, Brazil; anakarolinnesb@hotmail.com (A.K.d.S.B.); moraisgeovanni@gmail.com (G.d.M.L.); phumbertonunes@yahoo.com.br (P.H.M.N.); daniel.arcanjo@ufpi.edu.br (D.D.R.A.); 2 Department of Nutrition, Federal University of Piaui, SG-13, Ininga. Teresina, PI 64.049-550, Brazil; lmdefarias@yahoo.com.br (L.M.d.F.); lays_rosal@hotmail.com (L.A.R.L.R.); vanessablcarvalho@gmail.com (V.B.L.d.C.); karolfrota@ufpi.edu.br (K.d.M.G.F.); rayane_cm@hotmail.com (R.C.d.M.); 3 Department of Morphology, Federal University of Piaui. Teresina, PI 64.049-550, Brazil; cristian_cfcp@hotmail.com (C.F.d.C.P.); airton.conde@ufpi.edu.br (A.M.C.-J.); marciarizzo@ufpi.edu.br (M.d.S.R.); clarissembfonseca@gmail.com (C.M.B.F.); 4 Department of Nutrition, Federal University of Sergipe, São Cristóvão, SE 49100-000, Brazil; anamaraufs@gmail.com; 5 Biodiversity and Biotechnology Research Center, Federal University of Piaui, Parnaiba, PI 64202-020, Brazil; raimundaphb@gmail.com; 6 Faculty of Medicine, University of Brasília, Brasília, DF 70.910-900, Brazil; jrsaleite@gmail.com; 7 Department of Biophysics and Physiology, Federal University of Piaui, 64049-550. Teresina, PI 64.049-550, Brazil; marcosedfisio@gmail.com

**Keywords:** antioxidants, carotenoids, dyslipidemias, oxidative stress, psidium guajava

## Abstract

This work assessed the effects of a 28-day treatment with lycopene-rich extract (LRE) from red guava fruit (*Psidium guajava* L.) on the lipid profile and oxidative stress in an experimental model of dyslipidemia. Male hamsters (116.5 ± 2.16 g) were fed with the AIN 93G diet containing casein (20%), coconut fat (13.5%) and cholesterol (0.1%). The animals were divided into four groups: normolipidemic control (standard feed; NC, *n* = 7); hypercholesterolemic control (HC, *n* = 7); LRE 25 mg/kg/day (LRE-25, *n =* 7) and LRE 50 mg/kg/day (LRE-50, *n =* 9). After treatment, plasma concentrations of triglycerides (TG), total cholesterol (TC), low-density lipoprotein (LDL) cholesterol (LDL-c), high-density lipoprotein (HDL) cholesterol (HDL-c), malondialdehyde (MDA-p) and myeloperoxidase (MPO), as well as erythrocytic superoxide dismutase (SOD-e) and the atherogenic index, were determined. Malondialdehyde (MDA-h), catalase (CAT), glutathione peroxidase (GPx) and superoxide dismutase (SOD-h) levels were assessed. Feed intake (FI) and weight gain (WG) were also determined. The LRE-25 group presented significantly lower TG levels and atherogenic index than did the HC group (*p* < 0.05). Both LRE-25 and LRE-50 groups presented lower levels of MDA-p and MPO than did the HC group (*p* < 0.05). LRE demonstrated a promising effect against dyslipidemia and oxidative stress.

## 1. Introduction

Dyslipidemia is a clinical condition characterized by increased plasma concentrations of triglycerides and/or total cholesterol or fractions relative to reference values considered normal. Such alterations include hypertriglyceridemia due to the increase in the synthesis of very low-density lipoprotein (VLDL), reduction of the hydrolysis of triglycerides and/or hypercholesterolemia, resulting from the accumulation of cholesterol-rich lipoproteins, such as low-density lipoprotein (LDL). The increase in the lipids inside the vessels produces endothelial lesion. When associated with an inflammatory process, the lesion progresses to the formation of atherosclerosis plaques [1].

Considered an important cardiovascular risk factor, atherosclerosis is responsible for complications such as acute myocardial infarction and cerebrovascular stroke; most of these complications result from the rupture or erosion of atherosclerotic plaque and platelet aggregation, leading to thrombus formation [1,2]. The World Health Organization [3] estimates that in 2012 approximately 17.5 million people died from cardiovascular disease, accounting for 31% of all deaths worldwide.

Mechanisms that may trigger atherogenesis have not yet been fully elucidated. Among the hypotheses about its pathogenesis and progression, the involvement of oxidative stress is suggested due to its interruption of the balance between the production of reactive species and the antioxidant capacity [4]. It has been described that the oxidation of LDL is a key role factor to initiate the atherogenic process, since the presence of lipoperoxidation products can trigger oxidative modifications in LDL particles, inducing the degradation of apoliprotein B (apo B), an important structural protein that triggers inflammatory process and the formation of foam cells [5,6].

The cellular lesions produced by oxidative stress can be prevented or reduced by the action of antioxidant phytochemicals present in fruits and vegetables [7]. Lycopene is a lipophilic monounsaturated carotenoid responsible for the red color of some fruits and vegetables. It is present in foods such as tomato, guava, watermelon, papaya and *pitanga*, as well as in their derivatives [8,9]. Previous studies have demonstrated the beneficial effects of lycopene-rich extract (LRE), extracted from red guava (*Psidium guajava* L); Vasconcelos et al. [10] have reported the anti-inflammatory potential of the lycopene-rich extract from red guava in a carrageenan-induced acute inflammation model and Santos et al. [11] have reported that the lycopene-rich extract produced cytostatic and cytotoxic effects in breast cancer cells, as well as low cytotoxicity.

Considered to be an important antioxidant, lycopene is indicated as a possible cardiovascular protector by acting against oxidative damage in the endothelial cells [12]. McEneny et al. [13], after assessing the effects of lycopene on the function and modulation of high-density lipoprotein (HDL) in obese individuals, observed that this compound is able to reduce systemic inflammation and modulate the HDL phenotype into one that lowers atherogenic risk. Another study showed that the consumption of tomato products in rats, an important source of lycopene, attenuated liver steatosis, reduced the plasma lipoproteins associated to the atherogenic process and induced lipid metabolism [14].

Nonetheless, despite various studies that indicate the potential effect of lycopene on cardiovascular diseases [15,16], there are few studies evaluating the association of the reduction of cardiovascular events such as myocardial infarction, congestive heart failure, atrial fibrillation and atherosclerosis [17]. Thus, the present study aims to investigate the effects of a lycopene-rich extract from red guava fruit (*Psidium guajava* L.) on the lipid profile and oxidative stress markers in an experimental dyslipidemia model in hamsters.

## 2. Materials and Methods

### 2.1. Obtaining of the Lycopene-Rich Extract from Psidium guajava

The LRE was obtained from 500 g of fresh red guavas (*P. guajava* L.) at a high degree of maturation. They were submitted to extraction with ethanol, according to the methodology developed by Amorim, Leite and Ropke [18] and described in the patent no. BR102016030594-2. The content of lycopene in the LRE was determined by spectrophotometric analysis, indicating a content of 10 to 20% lycopene per dry extract. The LRE was freshly dissolved in 0.5% Tween80 in distilled water prior to oral administration in the hamsters.

### 2.2. Ethical Aspects

All procedures related to the use of animals were carried out according to the guidelines recommended by the “Guide for the Care and Use of Laboratory Animals” from the National Institutes of Health [19], with ethical principles recommended by the National Council for the Control of Animal Experimentation (CONCEA, Brazil), as well as by the Brazilian Laws (11,794 of 08.10.2008 and Law 9.605 of 12.02.98) [20,21]. The present study was approved by the Animal Experimentation Ethics Committee of the Federal University of Piauí (CEUA-UFPI No. 197/16).

### 2.3. Experimental Model of Dyslipidemia

Male hamsters (*Mesocricetus auratus,* Golden Syrian strain) (116.5 ± 2.16 g; 36 days-old) were kept in individual cages at a controlled temperature (23 ± 2 °C), 12-h light-dark cycle and free access to feed and water throughout the experiment. The hypercholesterolemic diet was specially elaborated for this study (PRAG Soluções Biociências, Jaú, SP, Brazil), and was composed of (in g/100 g of feed): casein (22.1); sucrose (5.0); starch (42.75); microcrystalline cellulose (10.0); soy oil (2.0); coconut fat (13.0); choline bitartarate (0.25); mineral mix AIN 93G (3.5); mix vit AIN 93G (1.0); and butylhydroxytoluene (BHT) (0.0024). Dyslipidemia was induced using a hypercholesterolemic diet for 21 days in all groups, except for the Normolipidemic Control (NC) group, which received standard rodent feed (normolipidemic diet; Labina, São Paulo, SP, Brazil) until the end of the experiment. This animal model was chosen since they are more susceptible to hypercholesterolemia induced by high-fat diets. A significant portion of their plasma cholesterol is linked to LDL, presenting metabolism that is similar to that observed in humans. Thus, this model is considered to be the most widely accepted to study the effects of diet on plasma lipid levels, as well as the mechanisms involved in this effect [22].

We determined the composition of standard normal and hypercholesterolemic feed, according to the Association of Official Analytical Chemists (AOAC) method [23]. Briefly, moistures were determined by heating in an oven at 105 °C until reaching constant weight. Mineral residues (ashes) were obtained by incineration of samples in a muffle furnace at 550 °C. For determination of lipid contents, a Soxhlet method was used, based on the weight loss of the material to be extracted with ethyl ether. Protein contents were determined by the Kjeldahl’s method, where the sample is digested using copper sulphate, potassium sulphate and sulfuric acid, and then submitted to distillation and subsequent titration with hydrochloric acid solution. Carbohydrate contents were calculated by the difference of the determined values for moisture, lipids, proteins and ashes. Total energy values were estimated by means of the energy conversion factors of Atwater: 4 kcal·g^−1^ for proteins, 4 kcal·g^−1^ for carbohydrates and 9 kcal·g^−1^ for lipids. Both diets possess similar levels of proteins and caloric total. The hypercholesterolemic feed presented a higher lipid content in relation to the normal feed. The moisture, ashes and carbohydrate contents were higher in the normolipidemic feed when compared with the hypercholesterolemic feed (Table 1).

After this period, the animals were treated for 28 days as follows: Normolipidemic Control (NC): animals that received standard feed; Hypercholesterolemic Control (HC): animals that received hypercholesterolemic feed; LRE-25 and LRE-50: animals that received hypercholesterolemic feed and LRE orally administrated at doses of 25 mg/kg/day or 50 mg/kg/day, respectively. During the period of treatment, measurement of body weight and calculation of feed intake were performed twice per week. 

**Figure 1:**
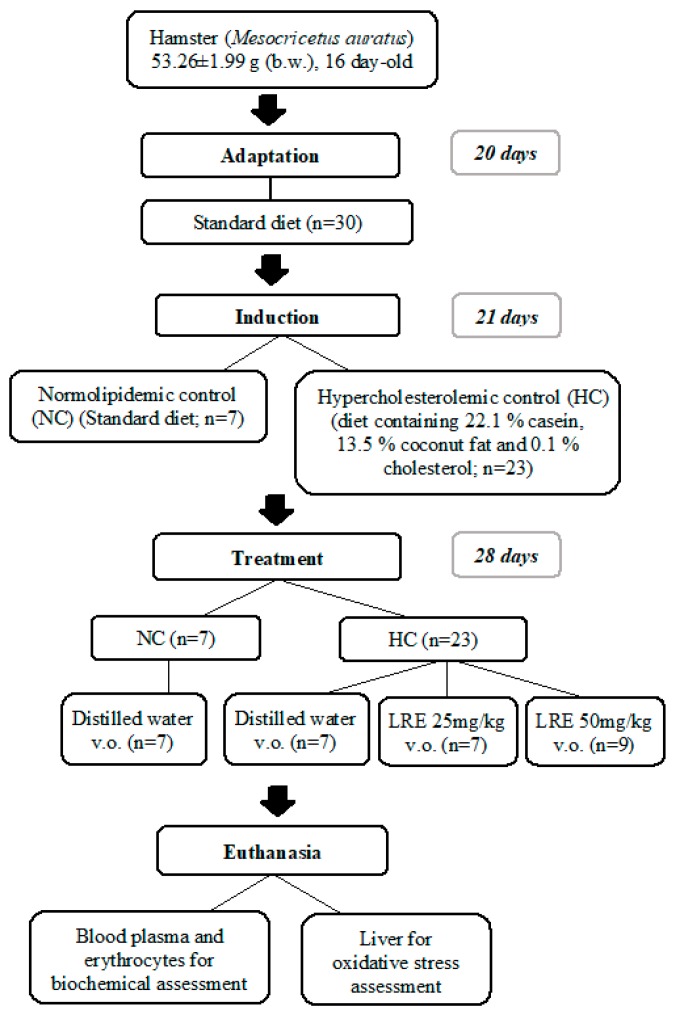
Scheme of experimental protocol of dyslipidemia induction and treatment with lycopene-rich extract (LRE) in hamsters.

At the end of treatment, the animals were euthanized after intraperitoneal administration of sodium thiopental (100 mg/kg per body weight) plus lidocaine (10 mg/kg). Venous blood was collected and centrifuged at 2500 RPM for 15 min at 4 °C (CIENTEC 4K15 Centrifuge) for the assessment of the lipid profile, oxidative stress biomarkers (malondialdehyde-MDA and myeloperoxidase-MPO) in the plasma. The cell content was washed three times with 5 mL of 0.9% saline solution followed by centrifugation, as stated above, for the determination of superoxide dismutase (SOD) activity in erythrocytes. Liver samples were also collected for the determination of MDA and the activity of the antioxidant enzymes Catalase (CAT), Glutathione Peroxidase (GPx) and Superoxide Dismutase (SOD) (Figure 1).

### 2.4. Determination of the Lipid Profile

The lipid profile (total cholesterol, HDL cholesterol, LDL cholesterol and triglycerides) was determined from plasma samples with the enzymatic colorimetric method in Labmax© Pleno biochemical analyzer using commercial colorimetric kits (Labtest Diagnóstica S.A., Lagoa Santa, MG, Brazil), according to the manufacturer´s instructions.

### 2.5. Atherogenic Indexes

The determination of atherogenic indexes was performed based on both the ratio between plasma levels of LDL-c and HDL-c, according to Draper et al. [24] and the ratio between plasma triglyceride levels and HDL-c, calculated as proposed by Dobiasova and Frohlich [25].

### 2.6. Determination of Oxidative Stress Biomarkers

#### 2.6.1. Determination of Plasma and Hepatic Concentrations of Malondialdehyde (MDA)

The MDA contents were determined by the production of thiobarbituric acid reactive substances (TBARS) according to the method described by Ohkawa et al. [26] with modifications. Briefly, 200 μL plasma, liver homogenate or distilled water were added to 350 μL of 20% acetic acid (pH 3.5) and 600 μL of 0.5% thiobarbituric acid. The mixture was incubated in a water bath at 100 °C for 45 min, and then cooled in an ice bath for 15 min. After this procedure, 50 μl of 8.1% sodium dodecyl sulfate (SDS) was added to the mixture and centrifuged for 15 min at 12,000 rpm at 25 °C. The absorbance was read at wavelengths of 532, 510 and 560 nm in a spectrophotometer. 

The supernatant was read at wavelengths of 532, 510 and 560 nm; the correct absorbance (ABS) was determined using the proposed formula in order to minimize the interference of heme pigments and hemoglobin: ABS = 1.22 × [A_532_ − (0.56 × A_510_) + (0.44 × A_560_)] [27]. An analytical calibration curve was prepared using MDA, as standard, at concentrations of 1, 5, 10, 25 and 50 nmol/mL. The results were expressed in nmol of MDA per ml of plasma (nmol/mL) or per g of liver homogenate (nmol/g liver).

#### 2.6.2. Determination of Plasma Activity of Myeloperoxidase (MPO)

The assessment of MPO activity was based on the oxidation rate of the substrate o-dianisidine in the presence of H_2_O_2_, which is determined by the change in absorbance measured at 450 nm [28]. Plasma samples were diluted in 1 mL of potassium phosphate solution (pH 6.0) and then centrifuged at 4500 rpm for 20 min at 4 °C. Absorbance levels were read on a microplate reader by adding the supernatant (10 μL) to the reading solution (200 μL), which was composed of distilled water, phosphate buffer (pH 6.0), 1.0% hydrogen peroxide and o-dianisidine. Monitoring of the rate of formation of o-dianisidine oxidation product was performed by observing the increase in the absorbance at 450 nm starting from the addition of the supernatant and after one minute. Results were expressed as unit of MPO per microliter of sample (U/μL).

### 2.7. Determination of the Antioxidant Enzyme Activity

#### 2.7.1. Liver Catalase (CAT) Activity

The CAT activity was determined according to the method described by Beutler [29]. The method is based on the quantification of the decomposition rate of hydrogen peroxide (H_2_O_2_) by CAT, which is measured by decreasing the optical density at 230 nm and 37 °C. The reaction was started by adding the liver homogenate (7.5 μL) in the reaction medium (250 μL) composed of 10 nM hydrogen peroxide, 1 mM Tris HCl buffer, 5 mM EDTA (pH 8.0) and Milli-Q© water. Samples were incubated at 37 °C and absorbance levels were read every 1 min up 6 min on the SynergyMx automatic plate reader at 230 nm. The results were expressed as U/mg protein. The concentration of total protein was determined using a commercial colorimetric kit, according to the manufacturer’s instructions (Labtest Diagnóstica S.A., Lagoa Santa, MG, Brazil). One enzyme unit (U) corresponded to the CAT activity able to hydrolyze 1 μmol of H_2_O_2_ per minute at 37 °C and pH 8.0.

#### 2.7.2. Liver Glutathione Peroxidase (GPx) Activity

The GPx activity was determined according to the method described by Sies [30], where GPx catalyzes the oxidation of glutathione by cumene hydroxide. Briefly, liver homogenate (10 μL) and 0.25 mM cumene hydroxide (5 μL) were added to the reaction medium (250 μL) composed of nicotinamide adenine dinucleotide phosphate (NADPH), Milli-Q© water, 5 mM EDTA, 0.1 U/mL reduced glutathione, 1.0 M glutathione and 0.1 M potassium phosphate buffer (pH 7.0). Absorbance levels were read in duplicate at 340 nm every minute up to 6 min using the SynergyMx automatic plate reader. The results were expressed as U/mg protein. One enzyme unit (U) corresponded to the GPx activity that was able to oxidize 1 μmol of NADPH per minute at 30 °C and pH 7.0.

#### 2.7.3. Liver Superoxide Dismutase (SOD-h) Activity

The SOD-h activity was determined according to the method described by McCord and Fridovich [31], based on the determination of superoxide anion produced by xanthine oxidase in the presence of xanthine. Briefly, liver homogenate (10 μL) and xanthine oxidase (7.5 μL) were added to the reaction medium (250 μl) composed of cytochrome C, Milli-Q© water, 500 μM xanthine, 1 mM EDTA and 0.05 M potassium phosphate buffer solution (pH 7.8). The blank sample was established with xanthine oxidase (7.5 μl) added to the reaction medium (250 μL). Absorbance levels were read at 550 nm every minute up to 6 min using a SynergyMx automatic plate reader. Samples were read in duplicate and the results were expressed as U/mg protein. One enzyme unit (U) corresponded to the SOD-h activity that was able to promote 50% inhibition of xanthine at 25 °C and pH 7.8.

#### 2.7.4. Erythrocyte Superoxide Dismutase (SOD-e) Activity

The SOD-e activity was determined according to the method described by Das, Samanta and Chainy [32], where the amount of SOD capable of 50% inhibition of nitrite formation was analyzed. Briefly, the reaction was initiated by the addition of the sample or phosphate buffer (blank) (100 μL) in a reaction medium composed of phosphate buffer, L-methionine, Triton X-100, hydroxylamine chloride and EDTA. Mixtures were incubated in a water bath at 37 °C for 5 min and after the addition of riboflavin (80 μL), they were exposed to light for 10 min. Finally, 1 mL of Griess reagent was added to the system and absorbance levels were read at 543 nm using a SynergyMx automatic plate reader. Determinations were made in duplicate and the results were expressed as U/g of hemoglobin. The concentration of hemoglobin was determined using a commercial colorimetric kit, according to the manufacturer’s instructions (Labtest Diagnóstica S.A., Lagoa Santa, MG, Brazil).

### 2.8. Statistical Analysis

The results were expressed as mean and standard error of the mean. For comparison of means among the groups, a variance analysis (ANOVA) was performed, followed by Tukey’s multiple comparison test, considering the significance level of 95% (*p* < 0.05). Graphs and statistical analyses were performed using the statistical software GraphPad Prism version 6.00 for Windows (GraphPad Software, La Jolla, CA, USA, www.graphpad.com). 

## 3. Results

### 3.1. Lipid Profile

**Figure 2:**
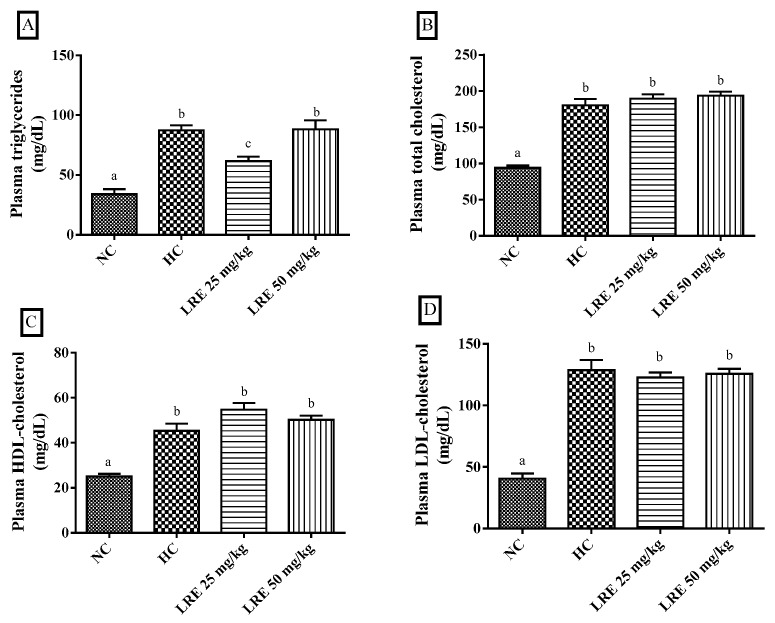
Plasma levels of triglycerides (TG) (**A**), total cholesterol (TC (**B**), high-density lipoprotein-cholesterol (HDL-c) (**C**) and low-density lipoprotein-cholesterol (LDL-c) (**D**) of hamsters submitted to experimentally induced dyslipidemia and treated with LRE. Each column represents mean ± SEM (standard error of the mean) of 7–9 animals/group. Two mean values that are not indicated with the same letter are significantly different from each other (*p* < 0.05). One-way analysis of variance (ANOVA), followed by Tukey’s test.

The LRE at 25 mg/kg promoted a significant reduction (*p* < 0.05) in plasma triglyceride (TG) levels (Figure 2A) when compared with HC. However, no significant differences were found regarding total cholesterol (TC) (Figure 2B), HDL cholesterol (HDL-c) (Figure 2C) and LDL cholesterol (LDL-c) (Figure 2D). On the other hand, the LRE at the dose of 50 mg/kg did not cause alterations in plasma levels of triglycerides, total cholesterol, LDL-cholesterol and HDL-cholesterol. The animals submitted to the hypercholesterolemic diet (HC) had serum triglycerides, total cholesterol, HDL cholesterol and LDL cholesterol levels significantly higher (*p* < 0.05) than did the animals submitted to the normolipidemic control (NC) diet.

### 3.2. Atherogenic Indexes

**Figure 3:**
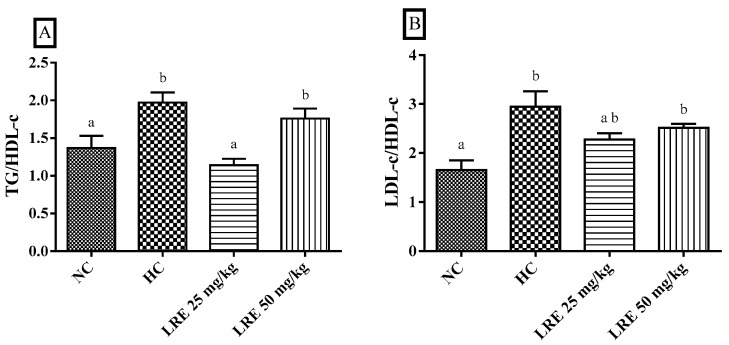
Atherogenic indexes of TG/HDL-c (**A**) and LDL-c/HDL-c ratios (**B**) of hamsters submitted to experimentally induced dyslipidemia and treated with LRE. Each column represents mean ± SEM (standard error of the mean) of 7–9 animals/group. Two mean values that are not indicated with the same letter are significantly different from each other (*p* < 0.05). One-way ANOVA followed by Tukey’s test.

The LRE at 25 mg/kg resulted in a reduction of the TG/HDL-c ratio (*p* < 0.05) when compared with animals with hypercholesterolemia (HC), but not of the LDL-c/HDL-c ratio. Interestingly, the atherogenic index of the HC group was increased when compared with the NC (Figure 3A,B).

### 3.3. Body Weight and Feed Consumption

**Figure 4:**
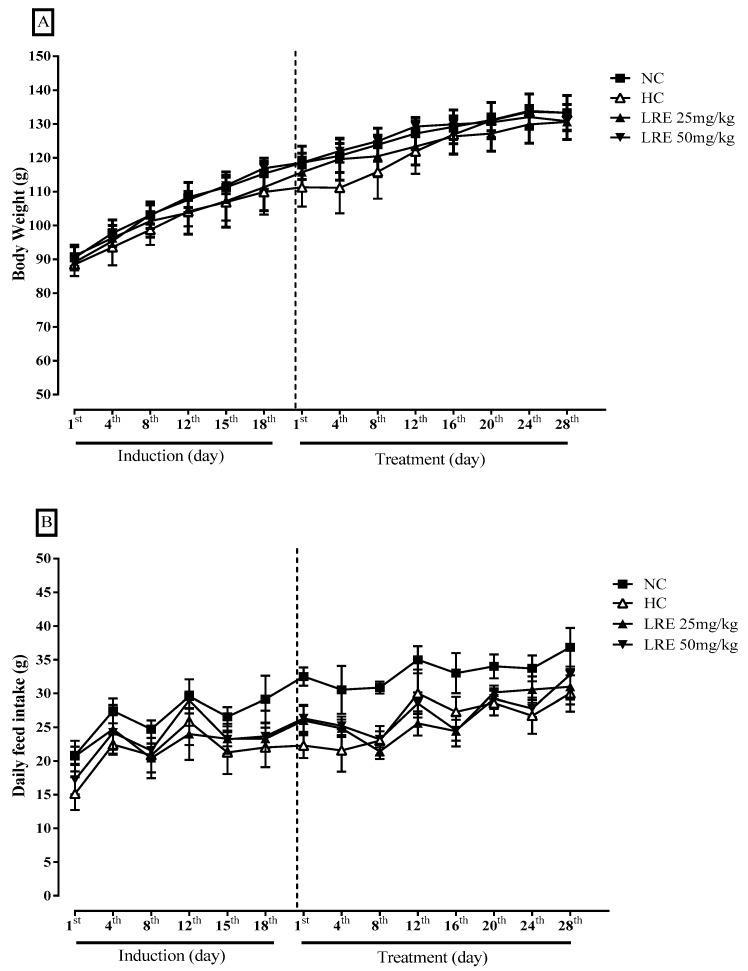
Body Weight (g) (**A**) and Feed Consumption (**B**) of hamsters submitted to experimentally induced dyslipidemia and treated with LRE. Results are expressed as mean ± SEM (standard error of the mean) of 7-9 animals/group.

No significant differences were observed between the groups in body weight of the animals (Figure 4A) or feed intake (Figure 4B) during the induction period of dyslipidemia and throughout the 28 days of treatment. 

### 3.4. Oxidative Stress Biomarkers

**Figure 5:**
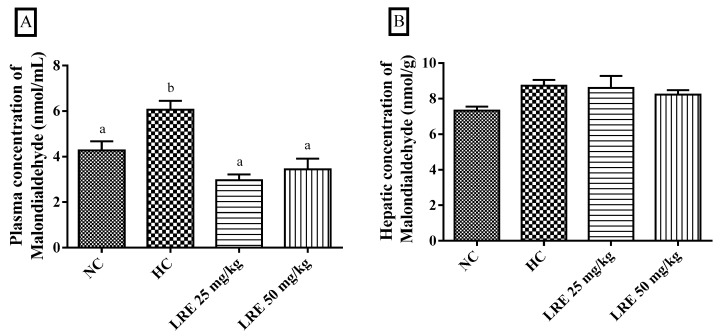
Concentration of malondialdehyde (MDA) in plasma (**A**) and liver (**B**) of hamsters submitted to experimentally induced dyslipidemia and treated with the lycopene-rich extract from LRE. Each column represents mean ± SEM (standard error of the mean) of 7-9 animals/group. Two mean values that are not indicated with the same letter are significantly different from each other (*p* < 0.05). One-way ANOVA followed by Tukey’s test.

The assessment of oxidative stress biomarkers MDA and MPO is shown in Figure 4. The LRE promoted a significant reduction of plasma malondialdehyde when compared with HC (*p* < 0.05) at doses of 25 and 50 mg/kg, indicating that the LRE was effective in reducing the dyslipidemia-related lipid peroxidation (Figure 5A). Besides, no significant differences were observed among groups in relation to malondialdehyde in liver tissue (Figure 5B).

**Figure 6:**
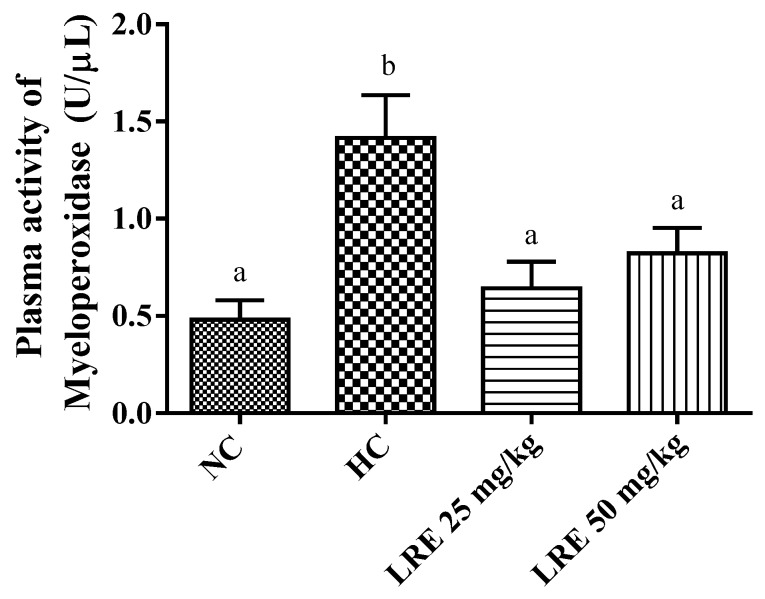
Plasma concentration of myeloperoxidase (MPO) of hamsters submitted to experimentally induced dyslipidemia and treated with LRE. Each column represents Mean ± SEM (standard error of the mean) of 7-9 animals/group. Two mean values that are not indicated with the same letter are significantly different from each other (*p* < 0.05). One-way ANOVA followed by Tukey’s test.

In this work, significantly lower values for MPO activity were observed in the groups treated with LRE at doses of 25 and 50 mg/kg when compared with HC. Besides, the induction of dyslipidemia with hypercholesterolemic diet (HC) promoted a significant increase in plasma MPO (*p* < 0.05) when compared with NC (Figure 6). These results indicate that the treatment with LRE promoted a decrease in the MPO activity related to the induction of hypercholesterolemia.

### 3.5. Activity of Antioxidant Enzymes

**Figure 7:**
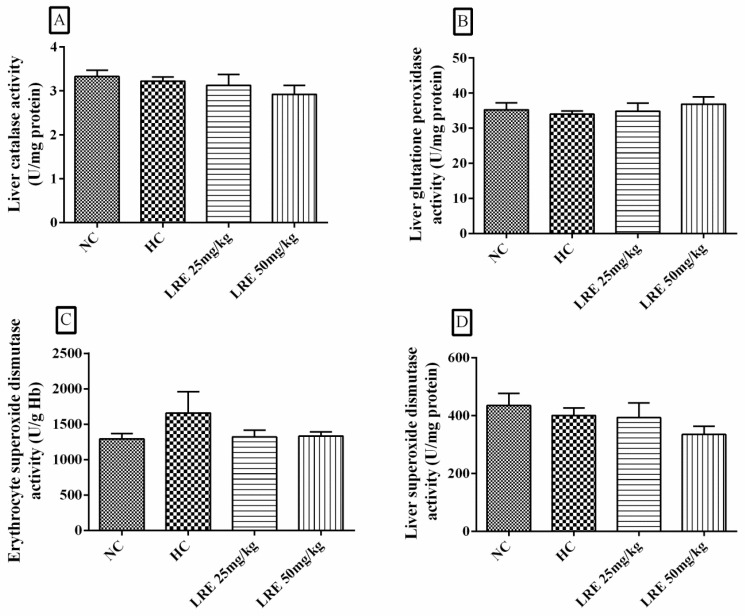
Activity of catalase (CAT), glutathione peroxidase (Gpx), erythrocytic superoxide dismutase (SOD-e) and superoxide dismutase (SOD-h) of hamsters submitted to experimentally induced dyslipidemia and treated with LRE. Each column represents mean ± SEM (standard error of the mean) of 7-9 animals/group.

The treatment with LRE at 25 and 50 mg/kg did not promote any alteration in the activities of CAT, GPx, SOD-h and SOD-e (Figure 7). Additionally, no significant differences were observed between the NC and HC groups, indicating that the induction of dyslipidemia did not induce significant changes in the activity of the antioxidant enzymes.

## 4. Discussion

The use of antioxidant compounds in the treatment of dyslipidemias has been increasing since several studies have suggested the involvement of oxidative stress in the pathogenesis and progression of atherosclerotic disease [4,33]. In this sense, the findings of this study suggest the protective effect of the LRE on a dyslipidemia model evidenced by the decrease in plasma levels of triglyceride and malondialdehyde, as well as the reduction of plasma myeloperoxidase activity and liver steatosis.

The high consumption of saturated fats is described as a risk factor for alterations in the lipid profile of humans and animals, which are associated with the formation and progression of atherosclerosis [1,34]. In our study, the LRE at the dose of 25 mg/kg was effective in lowering plasma TG levels. On the other hand, the same effect was not observed for the dose of 50 mg/kg. Similarly, Lee et al. [35] demonstrated the supplementation with tomato juice in hamsters submitted to a high-cholesterol diet and found that lycopene (2.787, 5.573 and 13.934 mg/kg) for 45 days was effective in reducing triglycerides. Kumar, Salwe and Kummarappan [36] also observed TG reduction in rats submitted to dyslipidemic diet and treated for 45 days with 10% lycopene formulation at a dose of 50 mg lycopene/kg body weight/day.

The hypotriglyceridemic effect of LRE may have positive repercussions in the treatment of dyslipidemias. Hypertriglyceridemia produces increased TG content in HDL and LDL, which increases the catabolism of HDL and induces the formation of small and dense LDL (which is more easily oxidized). In addition, hypertriglyceridemia increases the production of pro-inflammatory cytokines, fibrinogen and coagulation factors, compromising fibrinolysis and favoring the formation of thrombi in the vascular lumen [37]. 

LRE-mediated TG reduction may be related, at least in part, to the effect of lycopene on inhibiting lipogenesis and enhancing beta lipid oxidation. In this context, Fenni et al. [38] demonstrated that the treatment with purified lycopene (10 mg/kg feed/day) for 84 days in mice submitted to a diet-induced obesity promotes decrease in the expression of SREBP-1c (a transcription factor bound to a membrane that activates lipogenic genes) and the FASN gene (which encodes an enzyme that catalyzes the synthesis of fatty acids). The study also showed the effect of lycopene on increasing the induction of genes involved in fatty acid (FA) oxidation, such as: PPARα, a hepatic β-oxidation regulator; CPT-1, which facilitates the translocation of FA to mitochondria; and ACOX, which catalyzes the peroxisomal oxidation of FA. Thus, the lycopene present in the LRE might act by decreasing the availability of FA for TG formation, as well as by increasing the hydrolysis of TG, which would contribute to the reduction of its plasma levels.

On the other hand, Young and Lowe [39] have described that carotenoids may lose their efficacy as antioxidants at high concentrations or at high partial pressures of oxygen due to their autoxidation or the formation of carotenoid oxidation products. These products formed by the interaction with reactive species with high concentration of carotenoids are able to react with oxygen, forming the peroxyl radical that participates in peroxidation reactions. Thus, it is likely that LRE 50 mg/kg might have its effect decreased by the autoxidation and/or the formation of oxidized carotene byproducts from the dyslipidemia-related reactive species in animals. Then these species would induce alterations in the structure of lycopene, preventing its action.

In the present study, LRE did not promote alterations in plasma concentrations of total cholesterol (TC), LDL-cholesterol (LDL-c) and HDL-c. These results differ from those found in other studies using other formulations containing lycopene. Agarwal and Rao [40] point out that carotenoids such as lycopene and beta carotene may have an inhibitory effect on the enzyme HMG-CoA reductase of macrophages, a limiting enzyme in cholesterol synthesis. In fact, Alvi et al. [41] have demonstrated with in vitro studies that lycopene produces potent competitive inhibition of HMG-CoA reductase by binding to the hydrophobic moiety of the catalytic site of the enzyme.

In our study, a lycopene-rich extract containing around 10–20% lycopene was used, instead of the isolated compound, for treatments during a period of 28 days. Differently from the results found in this study, Lee et al. [35] used daily amounts of tomato juice, which are equivalent to the recommendation of lycopene for humans (22.5 mg/day) for 45 days in a model of dyslipidemia in hamsters, and observed a decrease in total cholesterol and LDL-c levels, without alterations of HDL-c. In a study with rats submitted to the induction of atherosclerosis with a cholesterol-rich diet, Renju et al. [42] treated animals with lycopene isolated from tomato at a dose of 10 mg/kg body weight for 60 days; a decrease in TC and LDL-c, as well as an increase in HDL-c, was observed. Thus, in this sense, LRE may contain other compounds that could possibly act to inhibit the action of lycopene, and that would explain in part the effects on these lipid parameters. Another aspect to be considered is the short period of treatment when compared to the other studies, which could have been insufficient to trigger an effect on these biochemical parameters.

The TG/HDL-c ratio is an important predictor of coronary events regardless of body mass index, which is considered as an accurate marker of insulin resistance and metabolic syndrome and is related to cardiovascular risks even when LDL is low [43]. In this study, the induction of hypercholesterolemia produced an increase in the TG/HDL-c ratio, which was reversed by the treatment with LRE at dose of 25 mg/kg. A study by Mohamed, Hamed and Al-Okbi [44] has also demonstrated a reduction of TG/HDL-c ratio in rats submitted to hypercholesterolemic diet and treated with a mixture of crude methanolic extract of celery seeds, rice bran oil and tomato acetone. From this perspective, the consumption of lycopene or lycopene-based products may be associated with reduced risk of vascular events.

From Define if appropriate another perspective, the atherogenic index obtained from the LDL-c/HDL-c ratio has been considered as a better indicator of risk for cardiovascular diseases when compared with individual parameters, which has been related to an increased risk of sudden cardiac death [45,46]. In a previous study, rats fed with a hyperlipidic diet and treated for 8 weeks with 8% lyophilized tomato paste, 24% lyophilized raw tomato or 0.1% mg pure lycopene showed a significant reduction of the LDL-c/HDL-c ratio [47]. Likewise, Salem [48] has also observed a reduced LDL-c/HDL-c ratio in rats fed with cholesterol-enriched food and supplemented with lycopene (350 mg/kg food). In the present study, no significant differences were observed among the LRE groups and CH in the LDL-c/HDL-c.

Regarding body weight, no significant differences were observed among groups. Likewise, no significant difference was observed among groups when dietary intake was evaluated. Similarly, Lee et al. [35] did not find differences in weight gain, as well as the intake of hamsters after hypercholesterolemic diet or the treatment with tomato juice. Considering that the feed consumption of the animals in the hypercholesterolemic group (HC) was similar to that found in the groups treated with the LRE, it could be inferred that the changes observed in the lipid profile were in fact derived from the treatment with the LRE, and from a lower consumption of lipids.

The excess plasma lipids observed in dyslipidemias may react with reactive species leading to lipid peroxidation [49]. In this process, compounds such as malondialdehyde (MDA), which cause cell damage, are formed [50]. In the present study, the two doses of the LRE produced reduced plasma concentrations of malondialdehyde when compared to those found in HC. Kumar et al. [37] also observed in lycopene-treated dyslipidemic rats a significant reduction in serum MDA levels compared to the untreated group and dyslipidemia. The decrease in the plasma concentration of MDA observed in the LRE-treated groups indicates that this extract was effective in reducing plasma lipid peroxidation increased by dyslipidemia.

Studies have suggested that lycopene may have a protective effect on reactive species such as hydroxyl and peroxynitrite [51,52]. Carotenoids can interact with reactive species in three distinct ways, namely: (1) by electron transfer reaction; (2) by removal of hydrogen; (3) by the addition of a radical species [53]. It is possible that the compounds present in the LRE reacted with the reactive species, reducing the production of oxidative damages in the plasma lipids, and thus, resulting in a lower production of MDA.

The myeloperoxidase (MPO) enzyme is largely produced during phagocytosis of LDLox particles, which increases the synthesis of reactive oxygen species and promotes a wider oxidation of lipoproteins [54,55]. In this sense, the present work showed an increase in MPO activity in the control group hypercholesterolemia (HC). It also showed that the treatments with the LRE produced a reversal of this increase. Similar results were described by Renju et al. [42] in a study with rats treated with lycopene 10 mg/kg body weight for 60 days; they presented lower MPO activity when compared to the hypercholesterolemia group in a model of atherosclerosis induced by a high-cholesterol diet. Using an experimental model of acute inflammation induced by carrageenan, Vasconcelos et al. [10] reported reduced MPO activity in peritoneal exudate of mice treated with purified lycopene obtained from the LRE.

The reduction of the myeloperoxidase activity mediated by the LRE points to the reduction of the oxidative stress induced by dyslipidemia, since the production of this enzyme during LDLox phagocytosis produces RE, which contributes to atherogenesis [54]. In addition, MPO is also involved in the lipid peroxidation process by its ability to induce the formation of new reactive species that are capable of removing hydrogen [56], contributing to the reduction of MDA levels, which corroborates the findings of this study.

The antioxidant enzymes act as a defense mechanism against oxidative damage, preventing and/or controlling the formation of RE; catalase (CAT) decomposes H_2_O_2_; superoxide dismutase (SOD) promotes the dismutation of O^2−^, while glutathione peroxidase (Gpx) acts by reducing organic hydroperoxides [57,58].

Regarding the activity of the antioxidant enzymes evaluated in this study, no change in enzyme activity was observed in either group, which indicates that the dyslipidemia induction model used did not produce inhibition or reduction of the activity of these enzymes. Differently, Renju et al. [42] observed that rats submitted to the induction of cholesterol-rich atherosclerosis had lower activity of liver enzymes CAT, SOD, GPx and glutathione reductase (GRd), whereas the treatment with lycopene 10 mg/kg body weight for 60 days produced an increase in the activity of these enzymes when compared to the group that consumed high-cholesterol diet. The methodological differences of this study in relation to those of Renju et al. [42] could account for, at least in part, the different results found. In this study, dyslipidemia was induced by the consumption of feed containing coconut fat (13.5%) and cholesterol (0.1%), with an experimental period of 49 days, while Renju et al. [42] used an induction model in rats with 10% corn oil, 1% cholesterol and 0.2% cholic acid - and the total duration of the experiment was 60 days.

However, studies indicate that lycopene is capable of inducing increased activity of antioxidant enzymes, including in models using healthy animals. Breinholt et al. [59] observed that in healthy rats receiving lycopene at concentrations of 1, 5, 50 and 100 mg/mL diluted in corn oil for 14 days, the lower concentrations produced an increase in the activity of the antioxidant enzymes SOD (1 and 50 mg/mL), GRd and GPx (5 mg/mL), while in the highest concentration the activity remained similar to that found in the control group, resulting in inverted dose-response curves in u-shape.

Based on the results of the abovementioned work, the lack of LRE effects on the antioxidant enzymes in this study may be related to the fact that the induction of the activity of these enzymes is within very restricted dose ranges, which may not have been achieved with the doses tested herein. This observation is particularly important when analyzing the lipid profile result, in which only the lowest dose had an effect on the TG reduction, suggesting that the LRE can also present an inverted “u” dose-response curve.

In addition, when evaluating the concentration of malondialdehyde in the liver tissue, it was observed that the treatments with the extract did not produce changes in relation to HC. In a different way, Renju et al. [42] found a decrease in hepatic MDA of mice treated for 60 days with lycopene (10 mg/kg body weight) obtained from tomato and the green algae *Chlorella marina*. The liver plays an important role in lipid metabolism because it is one of the organs responsible for the production of lipoproteins and fatty acids [60]. Therefore, the dysfunction of this organ can trigger in the accumulation of lipids, what contributes to the effects of lipid peroxidation. In addition, the fact that the 25-mg/kg dose of LRE had a better result compared to a 50-mg/kg dose reinforces the hypothesis that the LRE has an inverted “u” dose response curve.

The experimental model of induction of dyslipidemia using hypercholesterolemic feed in hamsters is well accepted. Although the inclusion of a standard diet without cholesterol and nutritionally equivalent to the hypercholesterolemic feed to the normal control group was not performed, significant differences in the lipid profile between normal and hypercholesterolemic control groups were observed, indicating that the induction of dyslipidemia was successful. On the other hand, no reduction or inhibition of antioxidant enzyme activity was observed. Therefore, it was not possible to determine the possible effect of the LRE on such enzymes. In spite of that, a reduction of triglycerides and oxidative stress markers after treatment with LRE was observed. A longer intake period of hypercholesterolemic feed might affect the antioxidant enzymes and allow studies in order to clarify the role of LRE in their activities.

The doses chosen for this study were determined based on Vasconcelos et al. [10], where the LRE was assayed in an experimental model of inflammation, and presented promising anti-inflammatory effects in rats. LRE at 25 and 50 mg/kg corresponds to doses of pure lycopene ranging between 2.5–5.0 mg/kg and 5.0–10 mg/kg, respectively. Moreover, even considering that the recommendation for human lycopene intake varies from 5.7 to 15 mg/day [61], as well as that lycopene supplementation has showed more promising results when compared to the consumption of foods containing lycopene in their composition [62], the presence of other different antioxidant compounds (e.g., carotenoids) in the LRE composition should be considered valuable [18,63]. Furthermore, new studies with long-period treatment could better elucidate the role of the LRE on antioxidant systems and oxidative stress.

## 5. Conclusions

The lycopene-rich extract from red guava fruit (*Psidium guajava* L.) promoted hypotriglyceridemic effect only at 25 mg/kg in an experimental model of dyslipidemia in hamsters. Besides, both doses of 25 and 50 mg/kg decreased plasma levels of lipid peroxidation biomarkers, evidenced by the reduction of plasma concentrations of MDA and MPO.

## Figures and Tables

**Table 1:** Composition of the standard diet and hypercholesterolemic diet.

Component	Normolipidemic Feed	Hypercholesterolemic Feed
Moisture (%)	10.2 ± 0.1	6.85 ± 0.17 *
Ashes (%)	7.8 ± 0.1	4.80 ± 0.19 *
Lipids (%)	3.3 ± 0.1	14.61 ± 0.07 *
Proteins (%)	20.9 ± 0.4	19.06±1.00
Carbohydrate # (%)	68.2 ± 0.3	61.30 ± 0.80 *
TEV (KJ.g^−1^)	16.36	19.00

Mean ± standard deviation. TEV—Total Energy Value. ^#^ Carbohydrate calculated by difference, including fibers. * *p* < 0.05 when compared with Normolipidemic Feed. Student’s *t*-test.

## Abbreviations

ACOX	Peroxisomal acyl-coenzyme A oxidase
AIN	American Institute of Nutrition
BHT	butylhydroxytoluene
CAT	catalase
FI	Feed intake
CPT-1	Carnitine palmitoyltransferase I
GPx	glutathione peroxidase
HC	hypercholesterolemic control
HDL-c	HDL cholesterol
HE	hematoxylin-eosin
HMG-CoA	3-hydroxy-3-methyl-glutaryl-coenzyme A
LDL-c	LDL cholesterol
LDLox	oxidized low-density lipoproteins
LRE	lycopene-rich extract
MDA-h	hepatic malondialdehyde
MDA-p	plasma malondialdehyde
MPO	myeloperoxidase
NADPH	nicotinamide adenine dinucleotide phosphate
NC	normolipidemic control
PPAR	Peroxisome proliferator-activated receptor
SDS	sodium dodecyl sulfate
SOD-e	erythrocytic superoxide dismutas
SOD-h	hepatic superoxide dismutase
SREBP	Sterol regulatory element-binding protein
TBARS	thiobarbituric acid reactive substances
TC	total cholesterol
TEV	Total Energy Value
TG	triglycerides
WG	weight gain
